# A multivariate meta-analysis of peripheral cytokine levels in obsessive compulsive disorder

**DOI:** 10.1192/j.eurpsy.2024.142

**Published:** 2024-08-27

**Authors:** Y. Chen, Q. Li, Y. Wang, Y. Wang, F. Long, F. Li

**Affiliations:** ^1^Huaxi MR Research Center (HMRRC), Department of Radiology, West China Hospital of Sichuan University; ^2^Research Unit of Psychoradiology, Chinese Academy of Medical Sciences, Chengdu, China

## Abstract

**Introduction:**

Obsessive-compulsive disorder (OCD) is a common psychiatric disorder. It is considered that dysregulation of cytokine levels is related to the pathophysiological mechanism of OCD. However, the results of previous studies on cytokine levels in OCD are inconsistent.

**Objectives:**

To perform a meta-analysis assessing cytokine levels in peripheral blood of OCD patients.

**Methods:**

We searched in PubMed, Web of Science, and Embase from inception to March 31, 2023 for eligible studies. We conducted multivariate meta-analysis in combined proinflammatory cytokines (interleukin-6 [IL-6], IL-1β, IL-2, tumor necrosis factor-α [TNF-α], and interferon-γ [IFN-γ]) and combined anti-inflammatory cytokines (IL-10 and IL-4) respectively, and calculated the same meta-analysis in each cytokine. We also performed sensitivity analysis and publication bias tests, as well as subgroup analysis (i.e. different age groups, varied cytokine measurement methods, medication treated or naïve, and presence of psychiatric comorbidities) and meta-regression analysis (variables including patients’ sex ratio, age, age at symptom onset, illness duration, scores of Y-BOCS, family history of psychiatric disorders, and BMI).

**Results:**

17 original studies (13, 13, 10, 5, 4, 3, 2 studies for IL-6, TNF-α, IL-1β, IL-10, IL-2, IL-4, and IFN-γ, respectively), 573 patients (mean age, 25.2; 50.3% female) and 498 healthy controls (HC; mean age, 25.3; 51.4% female) were included. The results showed that the levels of combined pro- or anti-inflammatory cytokines and each signle cytokine were not significantly different between OCD patients and HC (all *P*>0.05), with significant heterogeneities in all analyses (*I* ^2^ from 79.1% to 91.7%). We did not find between-group differences in cytokine levels in all subgroup analyses. Meta-regression analysis suggested that age at onset (*P*=0.0003) and family history (*P*=0.0062) might be the source of heterogeneity in TNF-α level. Sensitivity analysis confirmed that all results were stable, except for IL-4 where different cytokine measurement methods may be the contributing factor. Egger test did not find publication bias.

**Conclusions:**

Our study showed no difference in cytokine levels between OCD patients and HC, but age at onset and family history may affect TNF-α level. Confounding factors such as age at onset, family history, and cytokine measurement methods should be controlled in future studies to further explore the immune mechanism of OCD.

**Disclosure of Interest:**

None Declared

